# Investigating and Practicing Orthopedics at the Intersection of Sex and Gender: Understanding the Physiological Basis, Pathology, and Treatment Response of Orthopedic Conditions by Adopting a Gender Lens: A Narrative Overview

**DOI:** 10.3390/biomedicines12050974

**Published:** 2024-04-29

**Authors:** Carlo Biz, Rola Khamisy-Farah, Luca Puce, Lukasz Szarpak, Manlio Converti, Halil İbrahim Ceylan, Alberto Crimì, Nicola Luigi Bragazzi, Pietro Ruggieri

**Affiliations:** 1 Orthopedics and Orthopedic Oncology, Department of Surgery, Oncology and Gastroenterology (DiSCOG), University of Padova, 35128 Padova, Italy; albe.crim@gmail.com (A.C.); pietro.ruggieri@unipd.it (P.R.); 2 Azrieli Faculty of Medicine, Bar-Ilan University, Safed 1311502, Israel; rkhamisy@yahoo.com; 3 Clalit Health Service, Akko 2412001, Israel; 4 Department of Neuroscience, Rehabilitation, Ophthalmology, Genetics, Maternal and Child Health (DINOGMI), University of Genoa, 16132 Genoa, Italy; luca1puce@gmail.com; 5 Department of Clinical Research and Development, LUXMED Group, 02-676 Warsaw, Poland; lukasz.szarpak@gmail.com; 6 Henry JN Taub Department of Emergency Medicine, Baylor College of Medicine, Houston, TX 77030, USA; 7 Department of Mental Health, Local Health Unit ASL Napoli 2 Nord, 80027 Naples, Italy; manlioconverti@gmail.com; 8 Department of Physical Education of Sports Teaching, Faculty of Kazim Karabekir Education, Atatürk University, Erzurum 25030, Turkey; halil.ceylan@atauni.edu.tr; 9 Laboratory for Industrial and Applied Mathematics (LIAM), Department of Mathematics and Statistics, York University, Toronto, ON M3J 1P3, Canada; 10 Department of Food and Drugs, University of Parma, 43125 Parma, Italy

**Keywords:** sex and gender, orthopedics, personalized medicine

## Abstract

In the biomedical field, the differentiation between sex and gender is crucial for enhancing the understanding of human health and personalizing medical treatments, particularly within the domain of orthopedics. This distinction, often overlooked or misunderstood, is vital for dissecting and treating musculoskeletal conditions effectively. This review delves into the sex- and gender-specific physiology of bones, cartilage, ligaments, and tendons, highlighting how hormonal differences impact the musculoskeletal system’s structure and function, and exploring the physiopathology of orthopedic conditions from an epidemiological, molecular, and clinical perspective, shedding light on the discrepancies in disease manifestation across sexes. Examples such as the higher rates of deformities (adolescent idiopathic and adult degenerative scoliosis and hallux valgus) in females and osteoporosis in postmenopausal women illustrate the critical role of sex and gender in orthopedic health. Additionally, the review addresses the morbidity–mortality paradox, where women, despite appearing less healthy on frailty indexes, show lower mortality rates, highlighting the complex interplay between biological and social determinants of health. Injuries and chronic orthopedic conditions such osteoarthritis exhibit gender- and sex-specific prevalence and progression patterns, necessitating a nuanced approach to treatment that considers these differences to optimize outcomes. Moreover, the review underscores the importance of recognizing the unique needs of sexual minority and gender-diverse individuals in orthopedic care, emphasizing the impact of gender-affirming hormone therapy on aspects like bone health and perioperative risks. To foster advancements in sex- and gender-specific orthopedics, we advocate for the strategic disaggregation of data by sex and gender and the inclusion of “Sexual Orientation and Gender Identity” (SOGI) data in research and clinical practice. Such measures can enrich clinical insights, ensure tailored patient care, and promote inclusivity within orthopedic treatments, ultimately enhancing the precision and effectiveness of care for diverse patient populations. Integrating sex and gender considerations into orthopedic research and practice is paramount for addressing the complex and varied needs of patients. By embracing this comprehensive approach, orthopedic medicine can move towards more personalized, effective, and inclusive treatment strategies, thereby improving patient outcomes and advancing the field.

## 1. Introduction

In the biomedical arena, the concepts of sex and gender hold significant implications for understanding human health and the provision of medical care, and allow tailoring of treatments to improve patient outcomes [1]. However, these two terms are often used interchangeably or conflated, even if they carry distinct meanings that are essential to appreciate within the context of medicine and, in particular, orthopedics, a medical specialty dedicated to the study and treatment of musculoskeletal conditions. Analyses or sub-analyses disaggregating sex- and gender-related data are rarely conducted and reported, as shown by two comparative studies [2,3] that assessed research published in five and six leading, high-impact journals of orthopedic surgery, comprising two general and three/four subspecialty journals, respectively. Sex and gender are, indeed, overlooked in statistical models and the evaluation of the outcomes, with approximately only a third of the studies published incorporating sex and gender as factors in their analysis and identifying outcome differences between the sexes/genders.

To comprehensively address the issues related to sex and gender in orthopedics, it is essential to first differentiate between these terms and acknowledge their relevance in biomedicine. Sex refers to the biological attributes that distinguish individuals as male or female. It includes anatomical features and physiological characteristics, such as those related to reproductive organs, chromosomal patterns, and hormonal profiles. On the other hand, gender is a more complex construct embracing roles, behaviors, expectations, and identities that society attributes to individuals based on their perceived sex. While sex is often considered binary, with individuals classified as either male or female, even though the existence of intersex people may challenge this dichotomy, gender is a spectrum that encompasses diverse identities beyond the traditional binary framework [4].

Understanding the distinction between sex and gender is vital because it influences how orthopedic conditions are approached and treated. The musculoskeletal system, comprising bones, cartilage, ligaments, and tendons, plays a pivotal role in human mobility and overall well-being. In this article, we will delve into the physiology of bones and cartilage by applying a gender lens, explore the physiopathology of musculoskeletal conditions from different perspectives—from an epidemiological, molecular, and clinical standpoint—and discuss the implications of sex and gender in orthopedic treatment.

While there are systematic reviews with or without meta-analysis that cover specific aspects of orthopedics, to the best of our knowledge, there exists no review that provides a broad, comprehensive overview of the topic. Therefore, the present narrative overview was undertaken to fill in this knowledge gap.

## 2. Sex- and Gender-Specific Physiology of Bone

To better investigate and practice orthopedics at the intersection of sex and gender, it is crucial to first examine the fundamental physiological processes underlying human bone and cartilage. Bones, the rigid, mineralized structures that form the skeleton, serve multiple vital functions, providing support for the body, protecting internal organs, facilitating movement through articulations and joints, and serving as mineral reservoirs for calcium and phosphorus. From a macroscopic perspective, bones are divided into two forms: cancellous bones (also known as spongy or trabecular bone, about 20% of the entire skeletal mass) and cortical bones (or compact bone, approximately 80% of the skeletal mass). The former provides structural support and allows flexibility, while the latter provides protection and strength [5]. 

The composition and density of bones are influenced by sex-related hormonal variations, with site-specific actions. Indeed, estrogen plays a protective role in maintaining cortical bone density in both females and males and preserves cancellous bone density mostly in females, whilst testosterone and androgens affect periosteal growth in both sexes and bone remodeling mostly in males, specifically targeting trabecular bone turnover [6,7,8]. These variations seem to be age-dependent: indeed, in children before puberty, there are no reported differences in bone mineral content and bone mineral density between sexes. However, throughout puberty, females exhibit significantly greater bone mineral content and bone mineral density in the spine and pelvis compared to their male counterparts. In contrast, after puberty, males demonstrate higher bone mineral content and bone mineral density levels than females [6,7].

From a molecular perspective, testosterone is converted to highly active dihydrotestosterone by 5α-reductase in the cytoplasm of target cells, which binds to the androgen receptor present in chondrocytes and osteoblasts. Androgens (testosterone and dihydrotestosterone) can activate osteoblast precursors by initiating interleukin-1β (IL-1β) production, via binding to the androgen receptor, modulating its expression, or via insulin growth factor-1 (IGF-1) or transforming growth factor-β (TGF-β). Furthermore, testosterone regulates the expression of IGF-binding protein (IGF-BP). 

Testosterone can also be converted to estradiol (E2) by aromatase, exerting its estrogenic action by binding to the estrogen receptor (ER). There exist two main ER subtypes: namely, ER-α and ER-β, with ER-α being mainly associated with bone metabolism and turnover.

Both androgens and estrogens play crucial roles in reducing osteoblast cell death and promoting apoptosis in osteoclasts. Androgens indirectly lower osteoclast formation and longevity by affecting mesenchymal cells, while estrogens directly diminish nuclear factor kappa-B ligand, thereby obstructing osteoclast differentiation. Additionally, estrogens enhance osteoprotegerin output, altering the balance between osteoprotegerin and nuclear factor kappa-B ligand in favor of inhibiting osteoclast development [8,9,10,11]. Indeed, osteoprotegerin is a potent antiresorptive molecule that binds the final effector for osteoclastogenesis, the receptor activator of nuclear factor kappa-B ligand (RANK-L) [12]. Estrogens also reduce the levels of bone-resorbing cytokines, including interleukin-1 (Il-1), interleukin-6 (IL-6), and tumor necrosis factor-alpha (TNF-α) [13]. 

Key elements in this context are the Wnt/β-catenin signaling pathway and sclerostin, a secreted glycoprotein produced primarily by the osteocyte with anti-anabolic effects on bone formation. The Wnt pathway is essential for the final stages of osteoblast development and encourages osteoblasts to produce more osteoprotegerin [14]. Estrogen boosts osteoblast secretion of transforming growth factor beta, which, in turn, elevates Wnt production by osteoclasts. The bone’s Wnt pathway is suppressed by sclerostin and Dickkopf-related protein 1, with estrogen reducing the release of sclerostin by osteocytes [15]. In essence, sex hormones mainly decrease bone breakdown and support bone creation, playing a significant role in maintaining bone stability during adult life [8,9,10,11].

Furthermore, the regulation of bone matrix mineralization and vascularization appears to be sexually dimorphic, with some factors, like the bone-derived vascular endothelial growth factor (VEGF) [16], exerting distinct effects in males and females and resulting in divergent physical bone traits [17].

## 3. Sex- and Gender-Specific Physiology of Cartilage

Cartilage, a specialized connective tissue, is essential in orthopedics as it covers the ends of bones in articulations and supports structures like the nose, ears, and trachea. While the physiological differences between male and female cartilage are less pronounced than those of bone, hormonal fluctuations during the lifespan can impact cartilage health. For example, the decrease in estrogen during menopause may contribute to cartilage degeneration and joint issues in women. Overall, in adult populations, the thickness and volume of cartilage are significantly greater in males compared to females [18,19], as confirmed by a recently published systematic review of the literature pooling together eleven studies totaling 1962 patients (905 females and 787 males) [19]. While both sexes experience a decline in cartilage as they age, this reduction is more pronounced in females [18]. From a molecular standpoint, a study [20] utilizing a murine model found sex-specific differences in chondrogenic differentiation of muscle-derived stem cells in vitro and their ability to regenerate articular cartilage in vivo, with males showing superior chondrogenic differentiation and cartilage regeneration potential.

## 4. Sex- and Gender-Specific Physiology of Ligaments and Tendons

Tendons and ligaments play essential roles within the musculoskeletal system, providing connections and transmitting forces between muscle and bone, or bone and bone, and enabling locomotion [21]. However, the treatment of tendon-related disorders and injuries remains inadequately addressed. Specifically, the impact of estrogen- and androgen-like compounds on tendon biology has been generally overlooked in contemporary studies, even though sex hormones are widely recognized and significant in the physiology of other musculoskeletal components [22,23]. Using a murine model, a study [22] evaluated the mechanical properties, biochemical composition, transcriptome, and cellular activity of plantar flexor tendons. While the Achilles tendons of males were about 6% larger than those of females, the cell density of females was approximately 19% larger than that of males, without any significant differences in mechanical properties. At the mass spectrometry proteomics analysis, no significant difference between the two sexes in the abundance of major extracellular matrix proteins such as collagen type I and type III could be detected, even if females had about two-fold elevations in less abundant extracellular matrix proteins like fibronectin, periostin, and tenascin C. Finally, the transcriptome of male and female tendons was found to differ by only 1%.

## 5. Sex- and Gender-Specific Physiopathology of Orthopedic Conditions

Orthopedic conditions encompass a broad range of disorders affecting bones, cartilage, ligaments, tendons, and joints, ranging from deformities to injuries and cancers. These conditions can manifest differently based on an individual’s sex and gender, leading to variations in prevalence, severity, and progression (Table 1).

### 5.1. Sex- and Gender-Specific Physiopathology of Orthopedic Deformities

**Figure 1:**
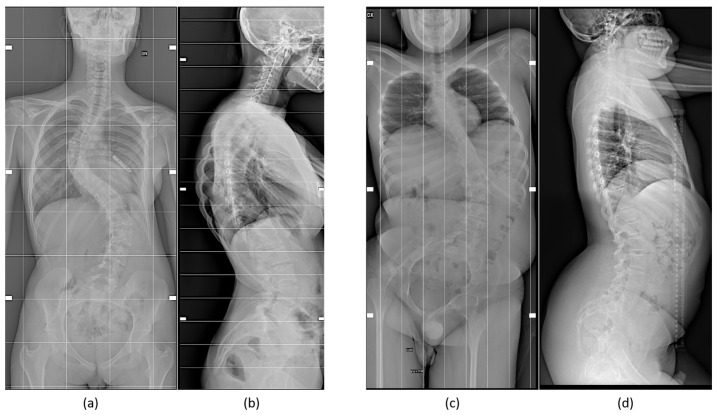
Female patient (**a**,**b**), 15 years old, with a severe thoraco-lumbar scoliosis, X-rays AP (**a**) and lateral (**b**) views. Idiopathic scoliosis has a known higher incidence rate in female patients. Male patient (**c**,**d**), 16 years old, with spinal muscular atrophy, X-rays AP (**c**) and lateral (**d**) views showing a severe lumbar scoliosis. Male patients usually have a lower incidence rate of scoliosis compared to female counterparts, apart from syndromic patients, but higher severity.

Some examples illustrating sex- and gender-specific differences include orthopedic deformities, such as adolescent idiopathic scoliosis (Figure 1), a condition that causes a curvature of the spine, typically arising during adolescence, and has an overall prevalence rate of 0.47–5.2%, occurring twice as frequently in females as in males [24,25], even if spine deformities are more severe in male subjects, as found in a retrospective study of 798 surgical patients, 140 boys and 658 girls, conducted in China [26].

Adolescent idiopathic scoliosis, a complex phenotype resulting from the interaction of different variables, related to spine anatomy and morphology [27], stiffness, growth velocity and curve patterns, and hormones, represents “one of the orthopedic disorders in which clinical evidence of sexual dimorphism is most marked” [25]. It is diagnosed around age 11–14 and 12–15 years in girls and boys, respectively [25]. 

Sex- and gender-related differences in post-operative pain perception in surgical patients with adolescent idiopathic scoliosis have been reported, with female patients undergoing posterior spinal fusion potentially benefiting from increased preoperative counseling and more aggressive intra- and post-operative pain management regimens [28].

Adult degenerative scoliosis exhibits sex- and gender-specific differences too, with the rigidness of spines being higher in males than in females and more pronounced in right than in left scoliosis, but only in male individuals [29]. However, another study [30] found that, while low back pain was more pronounced in male patients, scoliosis was more severe in females.

**Figure 2:**
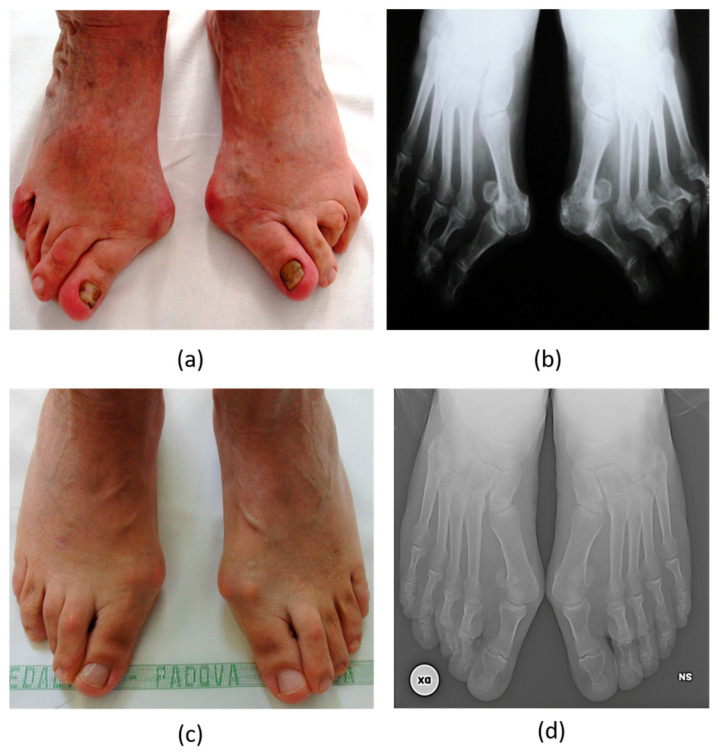
A 69-year-old woman (**a**,**b**) with evident forefoot deformities: (**a**) clinical image showing severe hallux valgus and lesser toe deformities including fixed claw toes and (**b**) weightbearing antero-posterior radiographic images showing the severe valgus of the big toe with osteoarthrosis of the metatarso-phalangeal joint, complete lateral sesamoid dislocation, and claw toes deformities of the lateral rays with dislocation of the metatarsal phalangeal joints on the left foot. A 71-year-old man (**c**,**d**) with forefoot pathologies: (**c**) clinical image showing moderate hallux valgus and some lesser toe deformities, including reducible claw toe deformities and (**d**) weightbearing antero-posterior radiographic images, showing the moderate hallux valgus without osteoarthrosis of the metatarso-phalangeal joint, sesamoid dislocations, and some claw toes deformities of the lateral rays without dislocation of the metatarsal phalangeal joints.

Also, hallux valgus, a condition that generates significant functional disability and foot pain, is more commonly found in women than in men (Figure 2). A systematic review and meta-analysis [31] that pooled together 78 papers reporting the results of 76 surveys, totaling 496,957 participants, found that, besides increasing with age, its prevalence was higher in females (30%, with a confidence interval spanning from 22% to 38%) compared to males (13%, with a confidence interval ranging from 9% to 17%). This higher rate in women might be attributed to the practice of wearing tight-fitting shoes and/or variations in bone structure. Research has, indeed, indicated that there are differences in the foot bones of males and females [31,32]. Moreover, sex/gender differences could be reported in post-operative outcomes, with male patients achieving greater correction of the hallux valgus angle than female patients, as shown in a retrospective study of 60 males (66 feet) and 70 females (82 feet) undergoing distal or proximal chevron osteotomy for the treatment of hallux valgus deformity between 2005 and 2011 in South Korea [32].

Differently from the previous conditions, Scheuermann kyphosis, also known as Scheuermann disease, juvenile kyphosis, or juvenile discogenic disease [33], is more commonly reported in males than in females [34], with a male to female ratio of at least 2:1. It is a developmental condition characterized by an exaggerated forward rounding of the upper back, diagnosed in adolescence, resulting from a structural abnormality in the vertebrae, where the front of the upper spine grows slower than the back, causing the vertebrae to become wedge-shaped and leading to the spine curving forward. 

Similarly, Legg–Calvé–Perthes disease (also known as Perthes disease), a rare childhood condition characterized by a temporary loss of blood supply to the femoral head, is mostly reported among male patients (with a gender ratio of 4:1). This condition causes joint pain and stiffness, and, over time, this lack of blood flow can cause the bone to become brittle and break easily, eventually leading to the reshaping of the hip joint as it heals. In females, the disease is uncommon, usually later in onset, and generally attributed to high-impact, repetitive athletics [35,36].

Finally, clubfoot (“talipes equinovarus”), a congenital condition where a newborn’s foot is twisted out of shape or position, appearing turned inward and downward, mostly affects males. This deformity, involving one or both feet, can significantly impair mobility but is often treatable through methods such as casting, bracing, or surgery to achieve a normal foot position and function. Gender ratios considerably vary from 1.6:1 to 2.5:1, even if, in some specific settings (i.e., in Australian Aborigines and in other Indigenous populations), ratios up to 4:1 have been reported, potentially for genetic reasons [37,38,39].

### 5.2. Sex- and Gender-Specific Physiopathology of Bone Loss

Other conditions are given by osteoporosis and osteopenia, which exhibit sex- and gender-specific rates, besides age-specific features [40]. Osteoporosis, characterized by decreased bone density and increased fracture risk, is more prevalent in postmenopausal women due to hormonal changes. Gendered expectations related to physical activity and nutrition can also influence the development of this condition. According to the US “National Health and Nutrition Examination Survey” (NHANES), the rate of osteoporosis in women who are 50 years or older is four times greater, and the occurrence of osteopenia is twice as high, compared to men of the same age group [41]. On the other hand, secondary osteoporosis, which is associated with pharmacological agents, including glucocorticoids (cortisone, prednisone, dexamethasone, and hydrocortisone), or underlying comorbidities like diabetes type 1 (lower bone strength), types 2 (increased bone cortical porosity), and other dysmetabolic conditions (the so-called “diabetic osteoporosis”), is more prevalent in men. Also, men have a higher risk of mortality following (either primary or secondary) osteoporotic fractures than women [42], as shown in the following section.

Renal osteodystrophy, a bone disorder resulting from chronic kidney disease/end-stage renal disease and encompassing various bone conditions that occur when the kidneys no longer function at a level needed for day-to-day life without dialysis or a kidney transplant, was investigated using a gender lens in a sample of 87 patients with chronic renal failure (44 males and 43 females, aged between 18 and 60 years) [43]. The findings revealed no substantial differences in biochemical marker levels between male and female patients at the same stage of chronic renal failure. However, an observed trend was the increase in intact parathormone and serum osteocalcin levels in women across both early and late stages of the disease. Bone changes were identified in 29 male patients (74.35%) and 25 female patients (60.97%), with a noted trend towards more frequent and severe bone alterations in men under 40 years of age. 

Beyond this age, the occurrence and intensity of bone changes were similar in both sexes. In conclusion, while bone alterations were more common and severe in men below 40 years of age, this pattern shifted post-menopause. Moreover, higher body weight positively affected bone changes only in females with advanced renal disease, with no such correlation observed in other patient groups.

### 5.3. Sex- and Gender-Specific Physiopathology of Orthopedic Injuries and Fractures 

Injuries represent another intriguing chapter of sex- and gender-specific orthopedics. According to a recently published current concepts review [44], encompassing 28 studies, sex- and gender-related differences could be identified in several key areas related to the management of total hip arthroplasty: namely, (i) the choice of surgical approach, use of robotic surgery, considerations of scar appearance, and selection of implants; (ii) outcomes and complications following surgery; (iii) the impact on sexual activity post-total hip arthroplasty; and (iv) psychological well-being and daily functional capabilities. Sex- and gender-specific analyses revealed that female patients may require more tailored considerations during the preoperative, operative, and post-operative stages to enhance clinical and functional results, minimize the likelihood of complications, and ensure patient satisfaction. The success of the total hip arthroplasty procedures was found to be significantly affected by factors related to sex and gender, which necessitates a careful evaluation and the adoption of specifically devised interventional strategies in surgical patients to enhance their satisfaction with the surgery’s outcomes and lower the incidence of sex-differentiated post-operative complications. 

Despite scoring higher on the frailty index, suggesting poorer health, including poorer bone and cartilage health, women experience lower mortality rates across all age groups, a phenomenon referred to as the “morbidity–mortality paradox”. This paradox, which is multifactorial and can be attributed to a set of anatomical, behavioral, and social factors and differences, is especially true for orthopedic fractures, including hip fractures [45]. The analysis of 512,715 adults aged 30–79 years from 10 regions in China, recruited between 2004 and 2008 and monitored over a decade, revealed sex- and gender-specific differences in the modifiable risk factors for hip fractures. While in men, low education, smoking, lower weight, alcohol consumption, and history of prior fracture were identified as the five major risk factors, contributing to 44.3% of hip fractures, in women, the significant risk factors were lower weight, low physical activity, diabetes, prior fracture, and self-reported poor health, accounting for 24.9% of hip fractures. While five modifiable risk factors were responsible for a significant proportion (approximately half) of hip fractures in men, they accounted only for a quarter in women. Two modifiable factors (lower weight and prior fracture) were shared between men and women.

From a clinical perspective, compared to men, women suffering from hip fractures are, indeed, less likely to undergo surgical treatment, whereas male sex/gender is a risk factor for facing post-operative complications and higher mortality rates [46,47]. A population-based cohort study [47], utilizing prospectively collected data from the “Danish Multidisciplinary Hip Fracture Registry”, identified 25,354 patients aged 65 years and older, 29% of whom were men. The 30-day mortality was computed at 15.9% for men and 9.3% for women, corresponding to an adjusted odds ratio of 2.30 (with a 95% confidence interval from 2.09 to 2.54). The overall readmission risk within 30 days after discharge was 21.6% for men and 16.4% for women (adjusted odds ratio of 1.38 (with a 95% confidence interval ranging from 1.29 to 1.47)), whereas no difference in the length of stay could be observed between the two sexes. Similarly, the mortality at one year was higher in men, as found in a retrospective study of 983 consecutive patients (206 males and 777 females) who sustained a nonpathological hip fracture [46].

**Figure 3:**
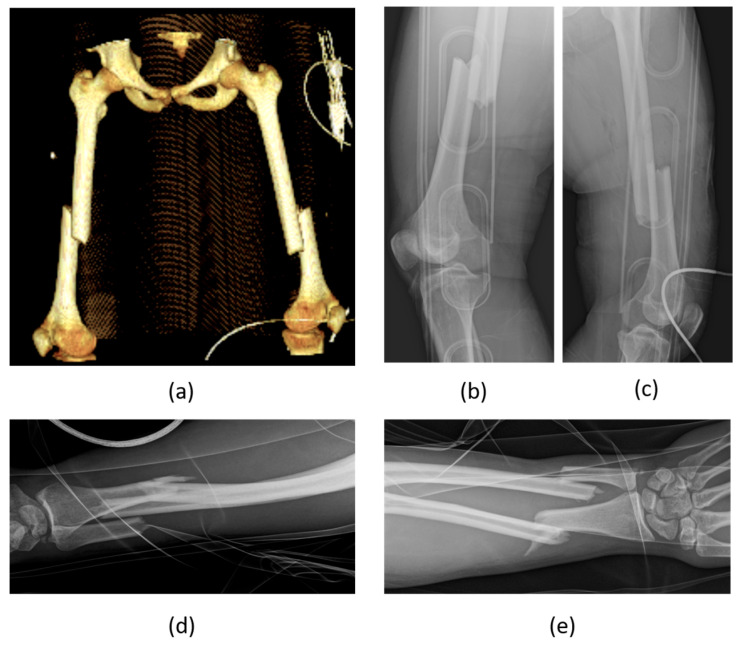
Young male patient, 20 years old, admitted for a polytrauma in an MVA (motor vehicle accident)—3D-CT reconstruction of the lower extremities with diaphyseal bilateral femur fractures (**a**), X-rays of the right femur (**b**), left femur (**c**), and left forearm fracture, lateral (**d**), and ap view (**e**). Young male patients are more commonly involved in MVAs compared to their female counterparts and trauma is one of the most common causes of death in this age class and gender.

A recently published, retrospective analysis of 16,359 cases from the “German Trauma Registry” [48] confirmed the “morbidity–mortality paradox” in the orthopedic setting. Women were found to be more commonly affected by pelvic fractures than men (with an incidence of 38.4/100,000 versus 33.4/100,000 among men). Of note, when categorizing the population into three age groups (young: under 35 years, middle-aged: 35–65 years, and elderly: over 65 years), it was observed that men were more prevalent in the younger two categories (Figure 3), while women were more common in the elderly group. Across these age groups, men reported a higher incidence of acetabular fractures, whereas women exhibited a higher incidence of unstable pelvic girdle fractures. Men also showed a higher rate of fractures caused by polytrauma. Furthermore, rates of surgery, morbidity, and mortality related to these injuries were higher in men compared to women.

Similarly to hip and pelvic fractures, also proximal humerus fractures, which account for 4–5% of all fractures, as well as other fragility fractures, are more commonly reported in the elderly female population, even if the mortality rate is consistently found to be higher in the male population [49].

### 5.4. Sex- and Gender-Specific Physiopathology of Sports Orthopedic Injuries

Anterior cruciate ligament tears, and specifically non-contact anterior cruciate ligament injuries, are more commonly reported among female athletes, possibly due to differences in joint structure, muscle activation and coordination, neuromuscular control, and, more broadly speaking, biomechanics [50,51]. According to a narrative review of the literature [51], female athletes demonstrate elevated quadriceps activity and reduced hamstring activity compared to their male counterparts. Enhanced hamstring activity was observed during selected injury risk situations, such as side-cutting or drop landings, with low medial hamstring activation and high vastus lateralis activation prior to landing being associated with an elevated incidence of anterior cruciate ligament injury. Gender-specific training programs, including preventive exercise-based intervention protocols on lower limb muscle activation, have been developed and deployed to mitigate this risk [50,51].

Female athletes are as well more susceptible to patellofemoral pain syndrome [52], often referred to as “runner’s knee”, a relatively common condition characterized by pain around or behind the patella (kneecap), mostly noticeable during activities that increase pressure on the knee joint, such as running, squatting, climbing stairs, or sitting for extended periods with bent knees.

Other sports injuries that are more common in female athletes include adhesive capsulitis or “frozen shoulder” [53], femoro-acetabular pincer-type impingement [54,55], rotator cuff tears [56], and shoulder instability [57], whilst femoro-acetabular cam-type impingement [58] mostly affects male athletes.

### 5.5. Sex- and Gender-Specific Physiopathology of Osteoarthritis

Osteoarthritis is another orthopedic condition with sex/gender-related disparities [59,60,61,62]. Osteoarthritis, a degenerative joint disease, affects both sexes but may progress differently. Hormonal factors, morphometric variables, such as joint anatomy, kinematics, and activity levels can contribute to variations in disease manifestation and progression [59,60,61]. 

Sex-specific anatomic differences included: (i) a larger discrepancy between the femorotibial angle and the hip–knee–ankle angle; (ii) narrower and smaller femurs with reduced femoral offset values but with a greater valgus neck-shaft angle; (iii) lower tibia morphometry measurements (mediolateral width and middle anteroposterior length); and (iv) hip morphometry measurements (lower acetabular dimensions and specific related angles, with higher lateral center edge angles and more frequent conditions like protrusio acetabuli).

Compared to men, besides exhibiting decreased cartilage volume, and smaller joint parameters and dimensions, from a clinical perspective, women appear to have higher osteoarthritis prevalence rates (61% versus 39%), use more healthcare resources, and report more clinical pain, inflammation, and physical difficulty [59].

From a molecular standpoint, a recent study [63] investigated a previously published knee articular cartilage transcriptome dataset comprising samples from 18 healthy individuals (5 females and 13 males) and 20 osteoarthritis patients (11 females and 9 males). Bioinformatics analysis identified 36 differentially expressed genes between healthy female and male cartilage samples, illustrating inherent sex-related molecular differences. Furthermore, 923 differentially expressed genes were found between osteoarthritis and healthy cartilage in females, enriching to 15 Reactome pathways, whereas in males, only 419 differentially expressed genes could be identified, with enrichment in six pathways. This disparity highlights a distinct osteoarthritis signaling response in male and female cartilage. Notably, 50 genes exhibited significantly different osteoarthritis-responsive expression changes between sexes, with 14 Reactome pathways such as “extracellular matrix organization” and “collagen biosynthesis and modifying enzymes” being particularly noteworthy among these sex-dependent osteoarthritis-responsive genes. 

Of note, sex/gender differences could be observed in the telomere length of leukocytes of 310 patients with knee osteoarthritis undergoing total knee arthroplasty versus 302 individuals free from the disease and in the cartilage of 57 osteoarthritis patients [64]. Telomere attrition is an important biomarker of ageing [65]. Findings revealed a notable interaction between sex and disease status concerning telomere length, with female patients exhibiting a significantly greater difference in leukocyte telomere length compared to their male counterparts, after adjusting for age.

Recently, sex- and gender-specific risk models for osteoarthritis have been proposed [66]. The study involved 10,958 participants from the Rotterdam Study who initially had no knee osteoarthritis in one or both knees. Of these, 1064 developed radiographic knee osteoarthritis over a median follow-up of 9.6 years. The link between each risk factor available and the incidence rate of the disease was assessed by using sex-specific multivariate regression models with generalized estimating equations, followed by statistical tests to identify sex-based differences in risk estimates. Subsequently, the population-attributable fractions were determined for modifiable risk factors. Results indicated that, overall, women had a higher prevalence of the risk factors under study, except for alcohol consumption and smoking, which were more prevalent in men, and high body mass index, which was equally prevalent in both sexes. Notable sex-specific differences in risk were observed: men had a higher relative risk for high levels of physical activity and a Kellgren and Lawrence score of 1 at baseline. In women, a body mass index equal to or greater than 27 kg/m^2^ was associated with a higher risk of developing radiographic knee osteoarthritis. The population-attributable fractions for higher body mass index were 25.6% and 19.3% in women and men, respectively, indicating a more pronounced impact of body mass index on women.

### 5.6. Sex- and Gender-Specific Physiopathology of Bone Infections

Differently from the previously analyzed conditions, males are more disproportionately impacted by bone infections [67], both in terms of susceptibility and response to infections. Osteomyelitis, characterized by an inflammatory condition affecting the bone and its marrow content, occurs more frequently in males (65–80%) compared to females (20–35%), with the highest incidence observed in the age group of 30–39 years [68].

Results from ten years of epidemiological surveillance of surgical site infections in Germany (between 2008 and 2017) [69] confirm a higher risk for male patients. Specifically, for orthopedics and traumatology (hip prosthesis following arthrosis, knee prosthesis, and arthroscopic procedures, but not for hip prosthesis following fracture) as well as abdominal surgery, the incidence rates were significantly higher for males, while for heart and vascular surgery, they showed opposite trends, being significantly higher for females.

Specifically concerning periprosthetic joint infection, a recent study [70] carried out a retrospective case-matched analysis of 156 patients in 78 pairs of males and females with staphylococcal infections (caused either by coagulase-negative staphylococci or Staphylococcus aureus) managed with two-stage exchange arthroplasty. There were no significant baseline differences by sex/gender except for greater use of chronic immunosuppression among females (16.4% versus 4.1%). Moreover, the three-year cumulative incidence of relapse tended to be higher for female patients (16.1% versus 8.8%), even though not in a statistically significant fashion.

### 5.7. Sex- and Gender-Specific Physiopathology of Orthopedic Malignancies

Osteoid osteoma, a common benign neoplasm, accounting for up to 3% of primary bone tumors, generally localized at the diaphysis or metaphysis of long bones of the lower limb, most frequently affects young male individuals, aged between 5 and 25 years [71].

Osteosarcoma, a bone tumor displaying a bimodal peak in incidence during the teen and late adult years [72], and soft tissue malignancies were found to be more common in male populations than in females across every race/ethnicity group, with an overall ratio of males to females computed at 1.3:1. Specifically, the incidence rate for males was 8.1 (with a 95% confidence interval of 7.7–8.6) compared to an incidence rate of 6.2 for females (with a 95% confidence interval of 5.8–6.6) [73].

**Figure 4:**
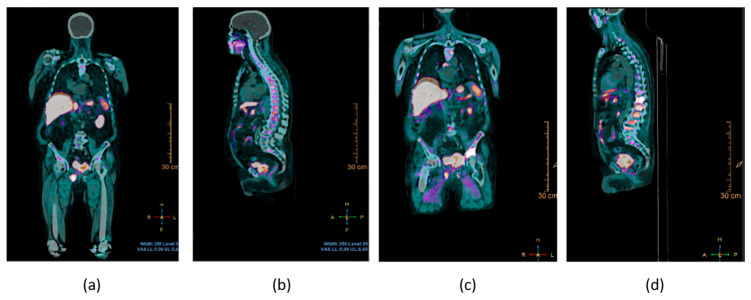
Male patient, 81 years old, with metastatic prostate cancer, choline PET-CT fusion images showing multiple bone lesions, coronal (**a**,**c**) and sagittal (**b**,**d**) reconstructions. Prostate cancer is the most common cancer in men; it can give bone metastasis but, due the osteosclerosing nature of these lesions, rarely requires orthopaedic surgery.

**Figure 5:**
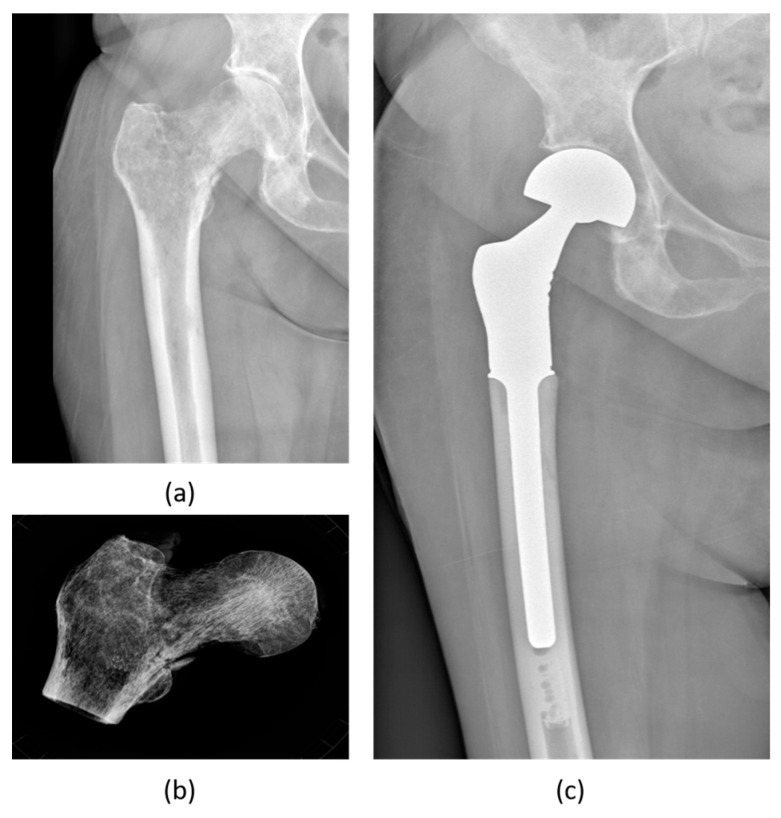
Female patient, 64 years old, with metastatic breast carcinoma, treated for a pathologic fracture of the proximal femur (**a**) with resection (**b**) and reconstruction with tumor prosthesis (**c**). Breast cancer is the most common cancer in women, often burdened by bone metastasis, occasionally presenting with a pathologic fracture.

Furthermore, female sex has been found to be associated with fewer bone metastases in various non-sex-specific cancers (1.8%, with a 95% confidence interval from 1.2% to 2.6%, in women versus 2.3%, with a 95% confidence interval ranging from 1.6% to 3.2% in men), with the effect being constant with changes in age and with a protective effect exclusively on the prognosis of respiratory system cancers [74]. However, the overall progression of the pain state induced by bone metastases was found not to differ between the two sexes [75] (Figure 4 and Figure 5).

Of note, surface osteogenic sarcoma is a cancerous condition that affects more women than men [76]. This tumor is a particular variant of classical osteosarcoma that develops on the outer surface of the cortex, instead of arising intramedullary [77], like parosteal osteosarcoma and periosteal osteosarcoma.

Finally, extra-abdominal fibromatosis is another oncological condition mostly reported among women [78], even if some investigations have found that female sex is not a risk factor [79].

### 5.8. Sex- and Gender-Specific Physiopathology of Tendinous Conditions

Women are more likely to report ligamentous laxity than men [80]. De Quervain’s tenosynovitis is a tendinous condition more commonly observed in women and is associated with pain and swelling in the first dorsal extensor sheath. A study [81] that utilized the large “Defense Medical Epidemiology Database” (DMED) analyzed the occurrence of de Quervain’s tenosynovitis from 1998 to 2006 through multivariate Poisson regression. Findings revealed 11,332 cases among a risk population of 12,117,749 person-years, with women experiencing a significantly higher incidence rate of 2.8 per 1000 person-years compared to 0.6 for men. Individuals over 40 years old had a rate of 2.0 per 1000 person-years, in contrast to 0.6 for those under 20 years of age. Moreover, ethnic disparities were evident, with black individuals having an incidence rate of 1.3 per 1000 person-years versus 0.8 for white individuals. 

Dupuytren’s disease, a fibroproliferative condition of the hand, generally affects males more commonly than females, even if the precise gender ratio varies according to the epidemiological setting—northern Europe, Australia, or the United States—reflecting the complex interplay of genetic and environmental factors across populations. The male-to-female ratios reported in studies range between 1.7:1 and 3:1, with studies reporting ratios up to 9.5:1 [82].

## 6. Sex- and Gender-Specific Response to Orthopedic Treatments

Understanding the interplay between sex/gender and outcomes is crucial for tailoring orthopedic treatments effectively. Physicians must consider not only the biological factors but also the psychosocial aspects related to gender identity and expression. Patients may have unique healthcare needs influenced by their gender and sex, which can impact treatment decisions, adherence, and outcomes.

For instance, orthopedic surgeons may need to modify instrumentations, surgical approaches, or rehabilitation protocols based on the patient’s sex-specific anatomical variations [83,84,85], even though some studies could not find any differences in responses to the intervention between the two sexes [86,87], which warrants further investigation.

Moreover, besides physical aspects, emotional ones should also be addressed. For example, not only physiologically, but also psychologically, women and men respond differently to pain and recovery, necessitating tailored pain management strategies that consider these differences. To improve treatment personalization, it is imperative that orthopedists collaborate with mental health professionals, considering as well the psychological impacts of chronic pain and physical disability associated with orthopedic conditions. This collaborative approach ensures that treatment plans are holistic and account for the interplay between physical health and emotional well-being, thereby enhancing recovery and patient satisfaction. Recommendations for orthopedic care must, therefore, emphasize the integration of gender-specific data into clinical decision-making processes to ensure that all patients receive care that is attuned to their unique psycho-physiological needs.

## 7. Orthopedic Management of Pregnant and Lactating Women

Pregnant and lactating/breastfeeding women are generally overlooked populations. Their orthopedic management requires careful consideration of both maternal and fetal health. During pregnancy and lactation, physiological changes in the body, including hormonal fluctuations and weight gain, can impact the musculoskeletal system, increasing the risk of certain conditions such as low back pain or carpal tunnel syndrome [88]. 

On the other hand, breastfeeding appears to be protective against osteoporotic hip fractures. According to a dose–response meta-analysis, pooling together seven studies of moderate-to-high quality, totaling 103,898 subjects, lactation can decrease their incidence rates, with a duration-dependent effect (the incidence tends to decrease with the increase in breastfeeding time, until 24 months, with no significant relationship when the duration exceeds 25 months) [89].

Treatment plans must be cautiously devised to avoid interventions that could harm the fetus or newborn, emphasizing non-invasive therapies and medications deemed safe during these periods. Surgical interventions are generally reserved for cases where non-operative treatments have failed or in emergencies, with timing and techniques adjusted to minimize risks. 

Close collaboration between orthopedic specialists, obstetricians, and pediatricians ensures the safety and well-being of both mother and child, making individualized care a priority to manage orthopedic conditions effectively during pregnancy and lactation [88].

## 8. Orthopedic Management of Sexual Minority Individuals

**Figure 6:**
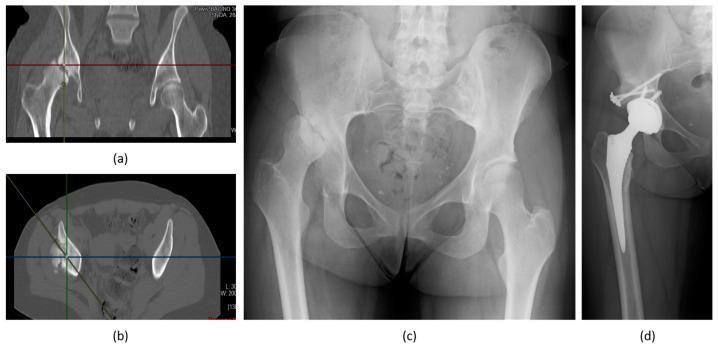
Female patient, 53 years old, with HIV, treated for acetabulum and femoral head AVN (avascular necrosis) with bone graft and total hip arthroplasty (THA). CT coronal (**a**) and axial (**b**) images, and X-rays ap view (**c**) showing the right femoral head and acetabulum AVN left untreated brought to a hip dislocation. Final treatment in the XR (**d**) showing bone grafting and THA. People living with HIV suffer from bone mineralization abnormalities, including osteoporosis and osteopenia; they can have AVN of the major joints.

Clinicians should be attentive to the needs of sexual minority individuals, including members of the LGBTQIA+ community and men having sex with men (MSM) [90,91]. These populations have been disproportionately impacted and are still impacted by the HIV pandemic. People living with HIV (PLWHIV) suffer from bone mineralization abnormalities, including osteoporosis and osteopenia [92] (Figure 6), the etiopathogenesis of which depends on various factors related to the host, the virus, and the specific regimen of antiretrovirals used, and their complex, non-linear interplay [93].

**Figure 7:**
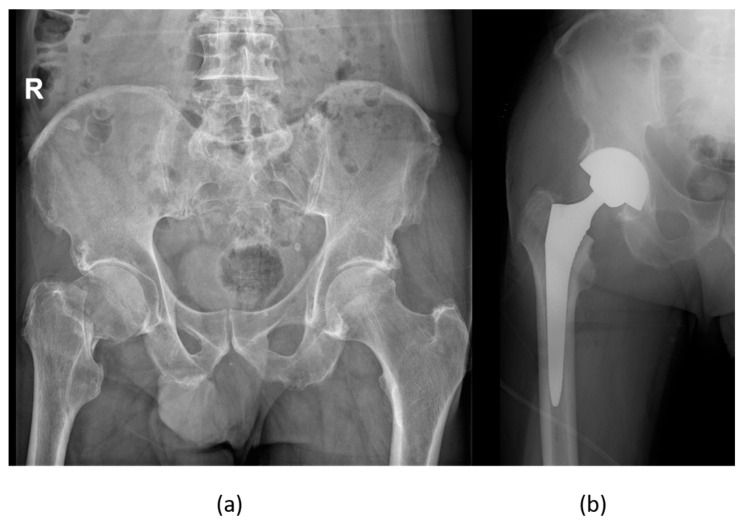
Male patient, 72 years old, with HIV treated for a femur neck fracture (**a**) with total hip arthroplasty (**b**), no post-operative complications, and good functional outcome. People living with HIV, thanks to the modern treatments, have a risk of infection around implants and rates of bone healing comparable to those in HIV-negative individuals.

Recent advancements in combined antiretroviral therapy (cART) have significantly changed the management of HIV infection, turning it into a manageable chronic condition that increases life expectancy. This shift means orthopedic surgeons are more likely to treat PLWHIV in their practices. Musculoskeletal symptoms are often seen in individuals with HIV, sometimes as the first sign of the infection, making it crucial for surgeons to be knowledgeable about related neoplasms and conditions affecting the muscles, bones, and joints for effective treatment. With the introduction of cART, procedures like total joint arthroplasty have become safer, though there remains a minor risk of perioperative infection, particularly in patients with uncontrolled HIV or CD4 counts below 400 cells/mm^3^. In trauma surgery, while the risk of infection around implants and the rates of bone healing in PLWHIV are similar to those in HIV-negative individuals (Figure 7), there is a heightened risk of complications such as pulmonary, renal, and infectious or septic issues in cases of severe trauma. For optimal patient care outcomes, factors including CD4 count, nutritional status, adherence to cART, viral load, and other conditions like hemophilia or infections from intravenous drug use must be considered when managing PLWHIV [93].

In the last decade, an innovative biomedical intervention, HIV pre-exposure prophylaxis (PrEP), involving the daily intake of antiretroviral medication, has been a true game-changer in the fight against HIV, being an extremely effective preventive strategy for people who are at high risk of HIV infection. HIV PrEP, when taken consistently, is able to significantly reduce their risk of contracting the virus. However, it is not without any side effects. According to a study [94], more than half (68%) of individuals who were prescribed HIV PrEP had bone risk factors, which should be taken into account when choosing an appropriate HIV PrEP regimen.

The impact of HIV PrEP on bone health depends on several factors, including (i) underlying bone risk factors prior to the initiation of the preventative strategy, (ii) HIV PrEP regimen adopted, (iii) adherence to the intervention, and (iv) duration of the intervention. Using linear mixed-effects models to estimate the bone mineral density decline in highly adherent PrEP users, a study [95] found that the percent decline in spine bone mineral density was monotonically associated with strata of increasing average weekly adherence (a 1.2% and a 0.5% drop in spine and hip bone mineral density, respectively). On average, this decrease was likely not clinically significant for most individuals on HIV PrEP, even though for those at the highest risk of fracture planning prolonged HIV PrEP use, alternative HIV PrEP strategies should be devised.

## 9. Orthopedic Management of Gender-Diverse Individuals

Furthermore, clinicians should be sensitive to gender-affirming care for transgender and gender-diverse individuals, respecting their identities and addressing any unique considerations in the treatment plan [96]. Gender-diverse or gender-expanded communities represent a highly marginalized, socially vulnerable population that consists of over 1.4 million individuals in the United States, with a prevalence being more pronounced among younger generations and being anticipated to rise significantly in the future. 

Gender-affirming hormone therapy influences, indeed, various physiological aspects relevant to orthopedic care, including bone health, the risk of fractures, and perioperative risks like venous thromboembolism and infections, are of paramount importance for orthopedic surgeons to consider.

A recent trial, the COMET study [97], collected an extensive set of parameters, spanning from clinical and laboratory data (such as phospho-calcic and hormonal blood tests and densitometric variables) to lifestyles (including smoking habits), in a sample of 125 transgender individuals (78 assigned females at birth and 47 assigned males at birth) before gender-affirming hormone therapy initiation. According to its findings, 14.3% of the transgender sample (versus 6.2% of controls matched by sex assigned at birth and age) reported low bone mineral density, which was found to be due to body composition and lifestyle factors.

However, at present, there is a paucity of studies on orthopedic needs in gender-diverse individuals and there are no studies specifically focusing on transgender individuals undergoing orthopedic surgeries within the broader field of orthopedic research, including joint arthroplasties [98], despite the medical comorbidities and risk factors often seen in this group, such as the risk of deep vein thrombosis associated with hormone therapy [99].

Orthopedic surgeons managing transgender individuals should carefully consider surgical positioning and address the need for social support post-surgery. Moreover, strategies to mitigate risks for those who may undergo gender reassignment surgery in the future involve the use of extended perioperative antibiotics and close monitoring for complications related to implant integration. Emphasizing comfort with and understanding of transgender patients is crucial to ensure they have equal access to healthcare services, with an accurate communication of surgery risks that are specific to them [99].

## 10. Role of Orthopedic Surgeons in Combating Gender-Based Violence

In the last decades, one in six women (16.0%) disclosed a history of gender-based violence within the past year, and one in three had even experienced intimate partner violence in their lifetime. While 1.7% of women attended an orthopedic fracture clinic as a direct consequence of the violence episode, only 14% of them had ever been asked about gender-based violence within a healthcare setting [100]. 

In recent years, non-pharmaceutical interventions, such as the social distancing measures and quarantine necessitated by the “Coronavirus Disease 2019” (COVID-19) pandemic, have led to a marked increase in reported instances of gender-based violence, including physical domestic violence against women [101]. 

Orthopedic surgeons can play a pivotal role in detecting victims of domestic violence through the identification of victim profiles and risk factors associated with such violence. Perpetrators are often intimate partners, including spouses or former spouses. Key risk factors for domestic violence include being a young adult woman, often belonging to visibly racialized communities and/or to lower socioeconomic strata, and being in short-term relationships. Injuries to the head and neck were the most common, with over a third of victims attributing their injuries to falls. Musculoskeletal injuries, noted in as many as 42% of cases, frequently involve the upper limbs and the torso. These injuries represent the primary cause of death among women aged 1 to 34 years [102].

Orthopedic surgeons can play a key role in the fight against domestic violence by leveraging their unique position to identify victims through specific injury patterns and risk factors. They can enhance their effectiveness through specialized training, the development of protocols for managing suspected cases, and by establishing connections with local support services. Furthermore, their involvement in documentation, referral, advocacy, and interdisciplinary collaboration can significantly contribute to the comprehensive care and support of victims, aiding in their recovery and protection.

## 11. Critical Observations and Practical Recommendations

To advance sex- and gender-specific orthopedics, there is a pressing need for comprehensive and detailed studies, including mechanistic analyses of biomarker differential expressions, molecular pathways, and OMICS profiling. Such research could steer preclinical and clinical investigations towards protocols that are tailored to address sex- and gender-specific differences [59].

Furthermore, it is advisable to systematically stratify and disaggregate data and outcomes by sex and gender, ensuring a more nuanced understanding of treatment effects. Additionally, collecting “Sexual Orientation and Gender Identity” (SOGI) data is crucial [103]. This approach not only enriches clinical insights but also tailors patient care, recognizing the diverse needs within patient populations. Through these practices, orthopedic care can become more inclusive and effective, reflecting the real-world diversity of patients, and enhancing treatment precision and outcomes for all.

Finally, sex and gender are rarely included and incorporated in orthopedic curricula [104], with students and residents in orthopedics showing insufficient sex- and gender-related competencies [105]. The exclusion of sex and gender considerations from orthopedic syllabuses has significant implications for both medical education and patient care. By not incorporating these factors into the educational framework, students and residents in orthopedics may graduate with a limited understanding of how sex and gender influence orthopedic conditions and treatment outcomes. This oversight can lead to several critical issues, including limited diagnostic skills, suboptimal treatment plans, reduced patient trust and satisfaction, negative impact on research and innovation, and reduced professional readiness and competence.

Addressing these issues requires a concerted effort to integrate sex and gender considerations into orthopedic curricula comprehensively. This would involve revising educational materials, incorporating case studies with diverse patient profiles, and promoting research that explores sex and gender differences in orthopedic conditions and treatments. Ultimately, such efforts can enhance the quality of orthopedic care and ensure it is equitable and responsive to the needs of all patients [106].

## 12. Conclusions

Osteoporosis and osteopenia, osteoarthritis, spinal disorders, and deformities, as well as fractures and other orthopedic conditions disproportionately impact females, even if males are more likely to face more severe and complicated conditions.

Investigating and practicing orthopedics at the intersection of sex and gender [107,108] requires an understanding of complex and multifaceted topics encompassing the physiological basis of bones and cartilages, the physiopathology of musculoskeletal conditions, and the response to orthopedic treatment [109,110], including emotional and psycho-social aspects. 

Recognizing and respecting the distinctions between sex and gender is paramount in providing holistic and individualized care. By considering these factors and including sexual minority and gender-diverse communities, orthopedic practitioners can better address the diverse needs of their patients, promoting better outcomes and improving the overall quality of musculoskeletal healthcare.

## Figures and Tables

**Table 1:** An overview of the sex- and gender-specific epidemiological burden of major orthopedic conditions.

Category	Condition/Disease	More Prevalent in
Deformities	Adolescent Idiopathic Scoliosis	Females, more severe in males
	Adult Degenerative Scoliosis	Mixed
	Hallux Valgus	Females
	Scheuermann Kyphosis	Males
	Legg-Calve-Perthes Disease (Perthes disease)	Males
	Clubfoot	Males
Bone Loss	Primary Osteoporosis	Females
	Secondary Osteoporosis	Males
	Osteopenia	Females
	Renal Osteodystrophy	Mixed age-dependent
Injuries and Fractures	Hip Fractures	Mixed, risk factors vary
	Total Hip Arthroplasty Complications	Mixed
	Pelvic Fractures	Mixed
	Proximal Humerus Fractures	Elderly Females
	Distal Radius Fractures	Females
Sports Orthopedic Injuries	Anterior Cruciate Ligament Tears	Females
	Patellofemoral Pain Syndrome	Females
	Adhesive Capsulitis (“Frozen Shoulder”)	Females
	Femoro-acetabular pincer-type impingement	Females
	Rotator cuff tears	Females
	Shoulder instability	Females
	Femoro-acetabular cam-type impingement	Males
Chronic Degenerative Condition	Osteoarthritis	Females
Bone Infections	Osteomyelitis	Males
Orthopedic Malignancies	Osteoid Osteoma	Young Males
	Osteosarcoma	Males
	Surface Osteogenic Sarcoma/Surface Osteosarcoma	Females
	Extra-Abdominal Fibromatosis	Females
Tendinous Conditions	Ligamentous Laxity	Females
	De Quervain’s Tenosynovitis	Females
	Dupuytren’s Disease	Males

## References

[1] White J., Tannenbaum C., Klinge I., Schiebinger L., Clayton J. (2021). The Integration of Sex and Gender Considerations into Biomedical Research: Lessons from International Funding Agencies. J. Clin. Endocrinol. Metab..

[2] Hettrich C.M., Hammoud S., LaMont L.E., Arendt E.A., Hannafin J.A. (2015). Sex-specific analysis of data in high-impact orthopaedic journals: How are we doing?. Clin. Orthop. Relat. Res..

[3] Gianakos A.L., George N., Pinninti A., Kwan S., LaPorte D., Mulcahey M.K. (2020). Sex- and Gender-specific Analysis in Orthopaedic Studies. Clin. Orthop. Relat. Res..

[4] Kaufman M.R., Eschliman E.L., Karver T.S. (2023). Differentiating sex and gender in health research to achieve gender equity. Bull. World Health Organ..

[5] Osterhoff G., Morgan E.F., Shefelbine S.J., Karim L., McNamara L.M., Augat P. (2016). Bone mechanical properties and changes with osteoporosis. Injury.

[6] Ortona E., Pagano M.T., Capossela L., Malorni W. (2023). The Role of Sex Differences in Bone Health and Healing. Biology.

[7] Mills E.G., Yang L., Nielsen M.F., Kassem M., Dhillo W.S., Comninos A.N. (2021). The Relationship between Bone and Reproductive Hormones beyond Estrogens and Androgens. Endocr. Rev..

[8] Narla R.R., Ott S.M. (2018). Bones and the sex hormones. Kidney Int..

[9] Compston J.E. (2001). Sex steroids and bone. Physiol. Rev..

[10] Almeida M., Laurent M.R., Dubois V., Claessens F., O’Brien C.A., Bouillon R., Vanderschueren D., Manolagas S.C. (2017). Estrogens and Androgens in Skeletal Physiology and Pathophysiology. Physiol. Rev..

[11] Shigehara K., Izumi K., Kadono Y., Mizokami A. (2021). Testosterone and Bone Health in Men: A Narrative Review. J. Clin. Med..

[12] Khosla S., Arrighi H.M., Melton L.J., Atkinson E.J., O’Fallon W.M., Dunstan C., Riggs B.L. (2002). Correlates of osteoprotegerin levels in women and men. Osteoporos. Int..

[13] Brylka L.J., Schinke T. (2019). Chemokines in Physiological and Pathological Bone Remodeling. Front. Immunol..

[14] Kim J.H., Liu X., Wang J., Chen X., Zhang H., Kim S.H., Cui J., Li R., Zhang W., Kong Y. (2013). Wnt signaling in bone formation and its therapeutic potential for bone diseases. Ther. Adv. Musculoskelet. Dis..

[15] Jia P., Zhang D., Zhang H., Wang A., Deng L., Xu Y. (2021). Bone Sclerostin and Dickkopf-related protein-1 are positively correlated with bone mineral density, bone microarchitecture, and bone strength in postmenopausal osteoporosis. BMC Musculoskelet. Disord..

[16] Hu K., Olsen B.R. (2016). Osteoblast-derived VEGF regulates osteoblast differentiation and bone formation during bone repair. J. Clin. Investig..

[17] Goring A., Sharma A., Javaheri B., Smith R.C., Kanczler J.M., Boyde A., Hesse E., Mahajan S., Olsen B.R., Pitsillides A.A. (2019). Regulation of the Bone Vascular Network is Sexually Dimorphic. J. Bone Miner. Res..

[18] Patel J., Chen S., Katzmeyer T., Pei Y.A., Pei M. (2023). Sex-dependent variation in cartilage adaptation: From degeneration to regeneration. Biol. Sex. Differ..

[19] Di Martino A., Barile F., D’Agostino C., Castafaro V., Cerasoli T., Mora P., Ruffilli A., Traina F., Faldini C. (2024). Are there gender-specific differences in hip and knee cartilage composition and degeneration? A systematic literature review. Eur. J. Orthop. Surg. Traumatol..

[20] Matsumoto T., Kubo S., Meszaros L.B., Corsi K.A., Cooper G.M., Li G., Usas A., Osawa A., Fu F.H., Huard J. (2008). The influence of sex on the chondrogenic potential of muscle-derived stem cells: Implications for cartilage regeneration and repair. Arthritis Rheum..

[21] Asahara H., Inui M., Lotz M.K. (2017). Tendons and Ligaments: Connecting Developmental Biology to Musculoskeletal Disease Pathogenesis. J. Bone Miner. Res..

[22] Sarver D.C., Kharaz Y.A., Sugg K.B., Gumucio J.P., Comerford E., Mendias C.L. (2017). Sex differences in tendon structure and function. J. Orthop. Res..

[23] Leblanc D.R., Schneider M., Angele P., Vollmer G., Docheva D. (2017). The effect of estrogen on tendon and ligament metabolism and function. J. Steroid Biochem. Mol. Biol..

[24] Konieczny M.R., Senyurt H., Krauspe R. (2013). Epidemiology of adolescent idiopathic scoliosis. J. Child. Orthop..

[25] Raggio C.L. (2006). Sexual dimorphism in adolescent idiopathic scoliosis. Orthop. Clin. N. Am..

[26] Wu Z., Zhu X., Xu L., Liu Z., Feng Z., Hung V.W.Y., Cheng J.C.Y., Qiu Y., Lee W.Y.W., Lam T.P. (2023). More Prevalent and Severe Low. Bone-Mineral Density in Boys with Severe Adolescent Idiopathic Scoliosis Than Girls: A Retrospective Study of 798 Surgical Patients. J. Clin. Med..

[27] Taylor J.R., Twomey L.T. (1984). Sexual dimorphism in human vertebral body shape. J. Anat..

[28] Collis R.W., Dry T., Chan G., Lim P., Oswald T. (2024). Sex related difference in postoperative pain and opioid use following posterior spinal fusion for adolescent idiopathic scoliosis. Spine Deform..

[29] Zheng J., Cheng B., Cook D., Yang Y. (2021). Gender differences in degenerative lumbar scoliosis spine flexibilities. Am. J. Transl. Res..

[30] Liang X., Yang P., Yuan H., Huo Y., Yang D., Wang H., Ding W. (2023). Sex-based differences in clinical and radiological presentation of patients with degenerative lumbar scoliosis: A cross-sectional study. J. Orthop. Surg. Res..

[31] Nix S., Smith M., Vicenzino B. (2010). Prevalence of hallux valgus in the general population: A systematic review and meta-analysis. J. Foot Ankle Res..

[32] Choi G.W., Kim H.J., Kim T.W., Lee J.W., Park S.B., Kim J.K. (2015). Sex-related differences in outcomes after hallux valgus surgery. Yonsei Med. J..

[33] Mansfield J.T., Bennett M. (2024). Scheuermann Disease. [Updated 2023 Jul 31]. StatPearls [Internet].

[34] Damborg F., Engell V., Nielsen J., Kyvik K.O., Andersen M.Ø., Thomsen K. (2011). Genetic epidemiology of Scheuermann’s disease. Acta Orthop..

[35] Guille J.T., Lipton G.E., Szöke G., Bowen J.R., Harcke H.T., Glutting J.J. (1998). Legg-Calvé-Perthes disease in girls. A comparison of the results with those seen in boys. J. Bone Jt. Surg. Am..

[36] Georgiadis A.G., Seeley M.A., Yellin J.L., Sankar W.N. (2015). The presentation of Legg-Calvé-Perthes disease in females. J. Child. Orthop..

[37] Kruse L.M., Dobbs M.B., Gurnett C.A. (2008). Polygenic threshold model with sex dimorphism in clubfoot inheritance: The Carter effect. J. Bone Jt. Surg. Am..

[38] Carey M., Bower C., Mylvaganam A., Rouse I. (2003). Talipes equinovarus in Western Australia. Paediatr. Perinat. Epidemiol..

[39] Chapman C., Stott N.S., Port R.V., Nicol R.O. (2000). Genetics of club foot in Maori and Pacific people. J. Med. Genet..

[40] Cawthon P.M. (2011). Gender differences in osteoporosis and fractures. Clin. Orthop. Relat. Res..

[41] Alswat K.A. (2017). Gender Disparities in Osteoporosis. J. Clin. Med. Res..

[42] Zhang Y.Y., Xie N., Sun X.D., Nice E.C., Liou Y.C., Huang C., Zhu H., Shen Z. (2024). Insights and implications of sexual dimorphism in osteoporosis. Bone Res..

[43] Kumchev E.P., Tzvetkova S.B., Enchev E.D., Yaneva M.P., Dimitrova R.H., Botushanova A.D., Dimitrakov D.J. (2000). Influence of age, sex and body weight on renal osteodystrophy in predialysis patients with chronic renal failure. Folia Med..

[44] Solarino G., Bizzoca D., Moretti A.M., D’Apolito R., Moretti B., Zagra L. (2022). Sex and Gender-Related Differences in the Outcome of Total Hip Arthroplasty: A Current Concepts Review. Medicina.

[45] Yao P., Parish S., Bennett D.A., Du H., Yang L., Chen Y., Guo Y., Yu C., Zhou G., Lv J. (2022). Gender differences in modifiable risk factors for hip fracture: 10-year follow-up of a prospective study of 0.5 million Chinese adults. J. Intern. Med..

[46] Endo Y., Aharonoff G.B., Zuckerman J.D., Egol K.A., Koval K.J. (2005). Gender differences in patients with hip fracture: A greater risk of morbidity and mortality in men. J. Orthop. Trauma..

[47] Kristensen P.K., Johnsen S.P., Mor A., Thillemann T.M., Pedersen A.B. (2017). Is the higher mortality among men with hip fracture explained by sex-related differences in quality of in-hospital care? A population-based cohort study. Age Ageing.

[48] Audretsch C.K., Siegemund A., Ellmerer A., Herath S.C. (2023). Sex Differences in Pelvic Fractures-a Retrospective Analysis of 16 359 Cases from the German Trauma Registry. Dtsch. Arztebl. Int..

[49] Pesce V., Vicenti G., Picca G., Rifino F., Carrozzo MMoretti B. (2016). A review of gender differences in proximal humerus fractures. J. Sex Gend. Specif. Med..

[50] Ellison T.M., Flagstaff I., Johnson A.E. (2021). Sexual Dimorphisms in Anterior Cruciate Ligament Injury: A Current Concepts Review. Orthop. J. Sport. Med..

[51] Bencke J., Aagaard P., Zebis M.K. (2018). Muscle Activation during ACL Injury Risk Movements in Young Female Athletes: A Narrative Review. Front. Physiol..

[52] Matzkin E., Garvey K. (2019). Sex Differences in Common Sports-Related Injuries. NASN Sch. Nurse.

[53] Erickson B.J., Shishani Y., Bishop M.E., Romeo A.A., Gobezie R. (2019). Adhesive Capsulitis: Demographics and Predictive Factors for Success Following Steroid Injections and Surgical Intervention. Arthrosc. Sport. Med. Rehabil..

[54] Owen M.M., Gohal C., Angileri H.S., Hartwell M.J., Plantz M.A., Tjong V.K., Terry M.A. (2023). Sex-Based Differences in Prevalence, Outcomes, and Complications of Hip Arthroscopy for Femoroacetabular Impingement: A Systematic Review and Meta-analysis. Orthop. J. Sport. Med..

[55] Okpala V.B., Tennent D.J., Johnson A.E., Schmitz M.R. (2018). Sexual Dimorphic Features Associated with Femoroacetabular Impingement. US Army Med. Dep. J..

[56] Razmjou H., Lincoln S., Macritchie I., Richards R.R., Medeiros D., Elmaraghy A. (2016). Sex and gender disparity in pathology, disability, referral pattern, and wait time for surgery in workers with shoulder injury. BMC Musculoskelet. Disord..

[57] Goodrich E., Wolf M., Vopat M., Mok A., Baker J., Bernard C., Tarakemeh A., Vopat B. (2021). Sex-specific differences in outcomes after anterior shoulder surgical stabilization: A meta-analysis and systematic review of literature. JSES Int..

[58] Zhou J., Melugin H.P., Hale R.F., Song B.M., Okoroha K.R., Levy B.A., Krych A.J. (2021). Sex differences in the prevalence of radiographic findings of structural hip deformities in patients with symptomatic femoroacetabular impingement. J. Hip Preserv. Surg..

[59] Tschon M., Contartese D., Pagani S., Borsari V., Fini M. (2021). Gender and Sex Are Key Determinants in Osteoarthritis Not Only Confounding Variables. A Systematic Review of Clinical Data. J. Clin. Med..

[60] Huber S., Mitterer J.A., Vallant S.M., Simon S., Hanak-Hammerl F., Schwarz G.M., Klasan A., Hofstaetter J.G. (2023). Gender-specific distribution of knee morphology according to CPAK and functional phenotype classification: Analysis of 8739 osteoarthritic knees prior to total knee arthroplasty using artificial intelligence. Knee Surg. Sport. Traumatol. Arthrosc..

[61] Astephen Wilson J.L., Dunbar M.J., Hubley-Kozey C.L. (2015). Knee joint biomechanics and neuromuscular control during gait before and after total knee arthroplasty are sex-specific. J. Arthroplast..

[62] Di J., Bai J., Zhang J., Chen J., Hao Y., Bai J., Xiang C. (2024). Regional disparities, age-related changes and sex-related differences in knee osteoarthritis. BMC Musculoskelet. Disord..

[63] Li C., Zheng Z. (2021). Males and Females Have Distinct Molecular Events in the Articular Cartilage during Knee Osteoarthritis. Int. J. Mol. Sci..

[64] Kuszel L., Trzeciak T., Begier-Krasinska B., Richter M., Li J., Czarny-Ratajczak M. (2024). Sex-specific differences in telomere length of patients with primary knee osteoarthritis. J. Cell Mol. Med..

[65] Bekaert S., De Meyer T., Van Oostveldt P. (2005). Telomere attrition as ageing biomarker. Anticancer Res..

[66] Szilagyi I.A., Waarsing J.H., Schiphof D., van Meurs J.B.J., Bierma-Zeinstra S.M.A. (2022). Towards sex-specific osteoarthritis risk models: Evaluation of risk factors for knee osteoarthritis in males and females. Rheumatology.

[67] Lener S., Wipplinger C., Hartmann S., Rietzler A., Thomé C., Tschugg A. (2021). Gender-Specific Differences in Presentation and Management of Spinal Infection: A Single-Center Retrospective Study of 159 Cases. Glob. Spine J..

[68] Calhoun J.H., Manring M.M. (2005). Adult osteomyelitis. Infect. Dis. Clin. N. Am..

[69] Aghdassi S.J.S., Schröder C., Gastmeier P. (2019). Gender-related risk factors for surgical site infections. Results from 10 years of surveillance in Germany. Antimicrob. Resist. Infect. Control.

[70] Higgins E., Tai D.B.G., Lahr B., Suh G.A., Berbari E.F., Perry K.I., Abdel M.P., Tande A.J. (2023). Sex-specific analysis of clinical features and outcomes in staphylococcal periprosthetic joint infections managed with two-stage exchange arthroplasty. J. Bone Jt. Infect..

[71] Napora J., Wałejko S., Mazurek T. (2023). Osteoid Osteoma, a Diagnostic Problem: A Series of Atypical and Mimicking Presentations and Review of the Recent Literature. J. Clin. Med..

[72] Mills L.J., Spector L.G., Largaespada D.A., Williams L.A. (2021). Sex differences in expression of immune elements emerge in children, young adults and mice with osteosarcoma. Biol. Sex. Differ..

[73] Cosci I., Del Fiore P., Mocellin S., Ferlin A. (2023). Gender Differences in Soft Tissue and Bone Sarcoma: A Narrative Review. Cancers.

[74] Ma W., Peltzer K., Qi L., Xu G., Liu Z., Wang J., Mao M., Chekhonin V.P., Wang X., Zhang C. (2019). Female sex is associated with a lower risk of bone metastases and favourable prognosis in non-sex-specific cancers. BMC Cancer.

[75] Falk S., Uldall M., Appel C., Ding M., Heegaard A.M. (2013). Influence of sex differences on the progression of cancer-induced bone pain. Anticancer Res..

[76] Nouri H., Ben Maitigue M., Abid L., Nouri N., Abdelkader A., Bouaziz M., Mestiri M. (2015). Surface osteosarcoma: Clinical features and therapeutic implications. J. Bone Oncol..

[77] Raymond A.K. (1991). Surface osteosarcoma. Clin. Orthop. Relat. Res..

[78] Asaad S.K., Abdullah A.M., Abdalrahman S.A., Fattah F.H., Tahir S.H., Omer C.S., Rashid R.J., Hassan M.N., Mohammed S.H., Kakamad F.H. (2023). Extra-abdominal recurrent aggressive fibromatosis: A case series and a literature review. Mol. Clin. Oncol..

[79] Cuomo P., Scoccianti G., Schiavo A., Tortolini V., Wigley C., Muratori F., Matera D., Kukushkina M., Funovics P.T., Lingitz M.T. (2021). Extra-abdominal desmoid tumor fibromatosis: A multicenter EMSOS study. BMC Cancer.

[80] Wilkerson R.D., Mason M.A. (2000). Differences in men’s and women’s mean ankle ligamentous laxity. Iowa Orthop. J..

[81] Wolf J.M., Sturdivant R.X., Owens B.D. (2009). Incidence of de Quervain’s tenosynovitis in a young, active population. J. Hand Surg. Am..

[82] Anthony S.G., Lozano-Calderon S.A., Simmons B.P., Jupiter J.B. (2008). Gender ratio of Dupuytren’s disease in the modern U.S. population. Hand.

[83] Fram B., Bishop M.E., Beredjiklian P., Seigerman D. (2021). Female Sex is Associated with Increased Reported Injury Rates and Difficulties with Use of Orthopedic Surgical Instruments. Cureus.

[84] Cho B.W., Nam J.H., Koh Y.G., Min J.H., Park K.K., Kang K.T. (2021). Gender-Based Quantitative Analysis of the Grand Piano Sign in Mechanically Aligned Total Knee Arthroplasty in Asians. J. Clin. Med..

[85] Moretti B., Spinarelli A., Varrassi G., Massari L., Gigante A., Iolascon G., Benedetti M.G., Moretti A.M. (2022). Influence of sex and gender on the management of late-stage knee osteoarthritis. Musculoskelet. Surg..

[86] Chang N.B., Bicknell R., Krupp R., Wiater J.M., Levy J., Athwal G.S. (2021). Sex-related differences in stemless total shoulder arthroplasty. JSES Int..

[87] Johnson A.J., Costa C.R., Mont M.A. (2011). Do we need gender-specific total joint arthroplasty?. Clin. Orthop. Relat. Res..

[88] Matthews L.J., McConda D.B., Lalli T.A., Daffner S.D. (2015). Orthostetrics: Management of Orthopedic Conditions in the Pregnant Patient. Orthopedics.

[89] Xiao H., Zhou Q., Niu G., Han G., Zhang Z., Zhang Q., Bai J., Zhu X. (2020). Association between breastfeeding and osteoporotic hip fracture in women: A dose-response meta-analysis. J. Orthop. Surg. Res..

[90] Chu A., Lin J.S., Moontasri N.J., Hammouri Q., Samora J.B. (2022). LGBTQ+ in Orthopaedics: Creating an Open and Inclusive Environment. J. Am. Acad. Orthop. Surg..

[91] Weaver D.J. (2022). What’s Important: Applying to Orthopaedic Surgery as a Member of the LGBTQ Community. J. Bone Jt. Surg. Am..

[92] Lima A.L., de Oliveira P.R., Plapler P.G., Marcolino F.M., de Souza Meirelles E., Sugawara A., Gobbi R.G., Dos Santos A.L., Camanho G.L. (2011). Osteopenia and osteoporosis in people living with HIV: Multiprofessional approach. HIV AIDS.

[93] Pretell-Mazzini J., Subhawong T., Hernandez V.H., Campo R. (2016). HIV and Orthopaedics: Musculoskeletal Manifestations and Outcomes. J. Bone Jt. Surg. Am..

[94] Fields S.D., Gruber J., Clue J., Rey G.G., Cuervo H.D. (2023). Prevalence of renal and bone risk factors among individuals prescribed oral pre-exposure prophylaxis for HIV. IJID Reg..

[95] Spinelli M.A., Glidden D.V., Anderson P.L., Gandhi M., McMahan V.M., Defechereux P., Schechter M., Veloso V.G., Chariyalertsak S., Guanira J.V. (2019). Impact of Estimated Pre-Exposure Prophylaxis (PrEP) Adherence Patterns on Bone Mineral Density in a Large PrEP Demonstration Project. AIDS Res. Hum. Retroviruses.

[96] Ramsey D.C., Lawson M.M., Stuart A., Sodders E., Working Z.M. (2021). Orthopaedic Care of the Transgender Patient. J. Bone Jt. Surg. Am..

[97] Ceolin C., Scala A., Dall’Agnol M., Ziliotto C., Delbarba A., Facondo P., Citron A., Vescovi B., Pasqualini S., Giannini S. (2024). Bone health and body composition in transgender adults before gender-affirming hormonal therapy: Data from the COMET study. J. Endocrinol. Investig..

[98] Harper K.D., Maiorino E. (2022). Total Joint Arthroplasties in Transgender Patients: Unique Considerations for an Emerging Patient Population. J. Am. Acad. Orthop. Surg..

[99] Bouck E.G., Grinsztejn E., Mcnamara M., Stavrou E.X., Wolberg A.S. (2023). Thromboembolic risk with gender-affirming hormone therapy: Potential role of global coagulation and fibrinolysis assays. Res. Pract. Thromb. Haemost..

[100] Sprague S., Bhandari M., Della Rocca G.J., Goslings J.C., Poolman R.W., Madden K., Simunovic N., Dosanjh S., Schemitsch E.H., PRAISE Investigators (2013). Prevalence of abuse and intimate partner violence surgical evaluation (PRAISE) in orthopaedic fracture clinics: A multinational prevalence study. Lancet.

[101] Kunasagran P.D., Mokti K., Ibrahim M.Y., Rahim S.S.S.A., Robinson F., Muyou A.J., Mujin S.M., Ali N., Chao G.G.C., Nasib R. (2024). The Global Landscape of Domestic Violence against Women during the COVID-19 Pandemic: A Narrative Review. Korean J. Fam. Med..

[102] Giordano V., Giordano C., Lopes I.M., Pires R.E., Godoy-Santos A., Giannoudis P.V. (2022). Orthopaedic surgeons can play important role in identifying victims of domestic violence in the emergency department—Narrative review of Brazilian literature. Medicine.

[103] Bragazzi N.L., Khamisy-Farah R., Converti M., Italian Working-Group on LGBTIQ Mental Health (2022). Ensuring equitable, inclusive and meaningful gender identity- and sexual orientation-related data collection in the healthcare sector: Insights from a critical, pragmatic systematic review of the literature. Int. Rev. Psychiatry.

[104] Miller V.M., Flynn P.M., Lindor K.D. (2012). Evaluating sex and gender competencies in the medical curriculum: A case study. Gend. Med..

[105] Matzkin E., Hu C.H., Gehrig L.B., Carter C. (2018). Does Sex Matter in Orthopedic Care?. Orthop. J. Harv. Med. Sch..

[106] Khamisy-Farah R., Bragazzi N.L. (2022). How to Integrate Sex and Gender Medicine into Medical and Allied Health Profession Undergraduate, Graduate, and Post-Graduate Education: Insights from a Rapid Systematic Literature Review and a Thematic Meta-Synthesis. J. Pers. Med..

[107] Bragazzi N.L., Bridgewood C., Watad A., Damiani G., McGonagle D. (2022). Sex-Based Medicine Meets Psoriatic Arthritis: Lessons Learned and to Learn. Front. Immunol..

[108] Sharif K., Omar M., Lahat A., Patt Y.S., Amital H., Zoabi G., Bragazzi N.L., Watad A. (2023). Big data- and machine learning-based analysis of a global pharmacovigilance database enables the discovery of sex-specific differences in the safety profile of dual IL4/IL13 blockade. Front. Pharmacol..

[109] Bryant J., Yi P., Miller L., Peek K., Lee D. (2018). Potential Sex Bias Exists in Orthopaedic Basic Science and Translational Research. J. Bone Jt. Surg. Am..

[110] Tosi L.L., Boyan B.D., Boskey A.L. (2005). Does sex matter in musculoskeletal health? The influence of sex and gender on musculoskeletal health. J. Bone Jt. Surg. Am..

